# Seizure classification in EEG signals utilizing Hilbert-Huang transform

**DOI:** 10.1186/1475-925X-10-38

**Published:** 2011-05-24

**Authors:** Rami J Oweis, Enas W Abdulhay

**Affiliations:** 1 Biomedical Engineering Department, Faculty of Engineering, Jordan University of Science and Technology, Irbid 22110, Jordan

## Abstract

**Background:**

Classification method capable of recognizing abnormal activities of the brain functionality are either brain imaging or brain signal analysis. The abnormal activity of interest in this study is characterized by a disturbance caused by changes in neuronal electrochemical activity that results in abnormal synchronous discharges. The method aims at helping physicians discriminate between healthy and seizure electroencephalographic (EEG) signals.

**Method:**

Discrimination in this work is achieved by analyzing EEG signals obtained from freely accessible databases. MATLAB has been used to implement and test the proposed classification algorithm. The analysis in question presents a classification of normal and ictal activities using a feature relied on Hilbert-Huang Transform. Through this method, information related to the intrinsic functions contained in the EEG signal has been extracted to track the local amplitude and the frequency of the signal. Based on this local information, weighted frequencies are calculated and a comparison between ictal and seizure-free determinant intrinsic functions is then performed. Methods of comparison used are the t-test and the Euclidean clustering.

**Results:**

The t-test results in a P-value < 0.02 and the clustering leads to accurate (94%) and specific (96%) results. The proposed method is also contrasted against the Multivariate Empirical Mode Decomposition that reaches 80% accuracy. Comparison results strengthen the contribution of this paper not only from the accuracy point of view but also with respect to its fast response and ease to use.

**Conclusion:**

An original tool for EEG signal processing giving physicians the possibility to diagnose brain functionality abnormalities is presented in this paper. The proposed system bears the potential of providing several credible benefits such as fast diagnosis, high accuracy, good sensitivity and specificity, time saving and user friendly. Furthermore, the classification of mode mixing can be achieved using the extracted instantaneous information of every IMF, but it would be most likely a hard task if only the average value is used. Extra benefits of this proposed system include low cost, and ease of interface. All of that indicate the usefulness of the tool and its use as an efficient diagnostic tool.

## Background

Electroencephalography (EEG) is an investigative method that provides information for the classification, diagnosis, and therapy of brain conditions. The frequency and energy content of EEG signals may contain helpful information about the nature of diseases affecting the brain.

In the past, physicians were asked to perform visual EEG analysis. To reduce the workload, computer programs for bio-signal analysis have been developed and used [1]. Since the first commercially available programs were introduced, computerized EEG analysis systems have become more sophisticated and less expensive with an increasing number of available programs.

The use of computerized EEG analysis has increased rapidly in health care. The information obtained via this computerized analysis is used to detect and diagnose normal and abnormal brain activities.

In patients with epilepsy, seizures occur suddenly. This brain disorder is a disturbance characterized by changes in neuronal electrochemical activity that results in abnormal synchronous discharges in a large cell population, which gives rise to clinical symptoms and signs. The computerized classification of epileptic seizures in EEG intracranial recordings is an important part in the epilepsy diagnostic procedure.

There are many variations and combinations of EEG features or parameters that can be measured, studied, analysed, and correlated one with et al. [2] have conducted a study to detect epilepsy using a linear approach based on main frequency, bandwidth and power. This approach yields good results for periodic signals. However, the accuracy of the method is based on the system of thresholds used for classification as well as on the nature of seizure. An alternative linear approach applied by Liu at al. [3] based on autocorrelation analysis has been performed to facilitate rhythmic activity tracking. In this study, regularity of spaced peaks of the same frequency has been characterized with the intention of rhythmic another and with other available data before a definite epileptic EEG analysis is made. Each of these features has its own sensitivity and specificity for classification of seizures. Qu seizure activity classification. Nonlinear methods applied to EEG dynamics have been also studied in other approaches to indicate changes in brain activity [4-6]. For instance, Pachori et al. [6] have implemented the mean frequency measure (centre of spectrum) as a feature of classification in order to identify the difference between ictal and seizure free intracranial EEG signals. The signal processing tool used for mean frequency calculation (Fourier-Bessel), though adapted to non-stationary signals, may not help in further important applications based on instantaneous frequency and amplitude tracking [7,8].

The outcomes of these studies called for efficient methods to perform seizure classification with accurate estimation of oscillatory information such as phase, frequency and amplitude. These are supposed to be an essential feature of comparison between ictal and seizure-free brain activities. A well-known method widely used to get such spectral information is the Hilbert Transform and its analytic signal representation [9,10]. In this context, this study comes with the aim of proposing a new method that relies on the coupling of Hilbert Transform and Empirical Mode Decomposition (EMD). This coupling is performed to extract information about the EEG signal intrinsic functions. The main properties of such an approach are adaptability to the non-stationary and non-linearity as well as to instantaneous (local) frequency and amplitude tracking. Hence, discrimination between healthy and seizure EEG signals using a weighted feature based on local oscillatory information is possible. One of the useful weighted features of Hilbert Transform is the Hilbert weighted frequency. It is anticipated that the outcome of this study leads to acquiring a tool allowing for the classification of abnormal activities of brain functionality. Since the EEG signals used in this paper are multivariate time series, comparison is performed between the frequency contents of the corresponding modes in seizure and seizure free signals. Contrast between EMD and Multivariate Empirical Mode Decomposition (MEMD), recently proposed by Rehman et. al and Rilling et. Al [11-13], is illustrated. This MEMD is capable of performing EMD on multivariate time series such that the corresponding modes have the same frequency content. Seizure detection in EEG signals utilizing MEMD is introduced in [12]. In [14], Tzallas et. al analyzed selected segments of EEG signals for seizure detection purposes using time-frequency and artificial neural network. Results of this work indicated an overall accuracy reaching 97.72%.

The work presented here is based on the results produced by the authors in [6] and [12]. This study when compared to [6] offers the possibility to track the instantaneous (local) frequency as well as the amplitude. By this tracking the weighted mean frequency, which is the main feature used to identify the abnormalities in EEG signals, is calculated accurately. Furthermore, authors in [6] use Fourier Bessel method, in which the selection of the optimum window size is required for good resolution. The fact that larger window size provides finer resolution in frequency comes at the expense of higher computation power (greater number of Fourier-Bessel coefficients). The proposed method also when measured up to [12], in which authors are facilitating decomposition simultaneously, offers higher accuracy by avoiding the mode mixing. This mode mixing makes the application of Hilbert transform to track the changes in the amplitude and frequency harder and thereby imposes the need for more time to complete the diagnostic procedure.

## Methods

In this study, the classification of abnormal activities of the brain functionality is achieved by understanding abnormal activities caused by changes in neuronal electrochemical activity through identifying the EEG signal features by utilizing Hilbert-Huang Transform. As indicated previously, EMD in this work is adapted to EEG signals [15]. Thus, it facilitated the extraction of the EEG intrinsic modes as well as the eventual EEG frequency/energy content analysis. The analysis of the frequency and energy content of every extracted mode has been performed via Hilbert Transform, which was achieved through the tracking of the instantaneous frequencies and amplitudes. Hilbert weighted frequency has been used to help discriminate between healthy and seizure EEG patterns.

### A. Hilbert Transform

The analytic signal z(*t*) of the real signal *x*(*t*), can be obtained from:(1)(2)(3)

where *a*(*t*) and *φ*(*t*) are the instantaneous amplitude and phase of *z*(*t*) respectively.

The instantaneous pulsation *ω*(*t*) of *z*(*t*) is expressed as:(4)

The Hilbert Transform *y(t*) of *x*(*t*) is given by [16]:(5)

The Hilbert weighted frequency is defined as [10]:(6)

This frequency () gives an idea about the mean frequency using instantaneous information (frequency *f *and amplitude *a *over an interval from the point index 1 to the point index k).

Many studies have successfully applied this method to wide-band neuronal signals. However, it has been shown that proper estimation of oscillatory parameters can be performed only on narrow-band signals. One of the methods that might be coupled to Hilbert Transform in order to get narrow-band signal is the nonlinear local technique known as Huang transform [17]. It can be used to adaptively represent the non-stationary signals as sums of zero-mean Amplitude Modulated-Frequency Modulated components called intrinsic mode functions (IMF).

### B. Empirical Mode Decomposition (Huang Transform)

**Figure 1:**
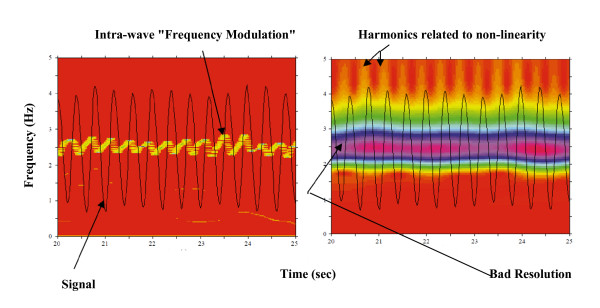
**Comparison between Hilbert-Huang Transform (left) and wavelet Transform (right)**. Comparison between Hilbert-Huang Transform (left) and wavelet Transform (right) when applied to the same signal (black). Frequency modulation is tracked precisely by Hilbert-Huang (yellow curve) while the resolution constraints and resultant harmonics prevent accurate tracking of frequency modulation by wavelet transform.

EMD is a signal processing technique used to extract all the oscillatory modes embedded in a signal without any requirement of stationarity or linearity. The extracted modes, with well-defined instantaneous frequencies (accurate estimation of oscillatory information), are speculatively associated with specific physical aspects of the phenomenon investigated. When compared to wavelet decomposition, which is a wavelet-model based method, EMD is a data driven method, has no resolution or harmonics complications as indicated in Figure 1. The investigation of Figure 1 makes it evident that the results obtained by Hilbert-Huang Transform overcome the disadvantages induced by the wavelet transform. Figure 1 illustrates that the instantaneous local frequency/amplitude information cannot be precisely extracted by the wavelet transform. It also shows that Hilbert-Huang spectrum, which is the application of Hilbert Transform to the extracted modes, is having high accuracy in this direction.

By definition, an Intrinsic Mode Function (IMF) satisfies two conditions. These are: a) The number of extrema and the number of zero crossings may differ by no more than one. b) The local average, defined by the average of the maximum and minimum envelopes, is zero. These properties of intrinsic mode allow for defining the instantaneous frequency and amplitude in an unambiguous way. Hilbert Transform can then be applied to every single intrinsic mode.

Given these two defining requirements of an intrinsic mode, the sifting process for extracting intrinsic modes from a given signal *x(t)*, *t *= 1,..., *T *can be implemented by the following procedure:

1. Identify all the maxima and minima of *x(t)*,

2. Generate its upper and lower envelopes, *X*_*up*_(*t*) and *X*_*low*_(*t*), with cubic spline interpolation,

3. Calculate the point-by-point mean from the upper and lower envelopes, by using *m(t) *= (*X*_*up*_(*t*) *+ X*_*low*_(*t*))/2,

4. Extract the detail, *d(t) *= *x(t) *- *m(t)*,

5. Test the following two conditions of *d(t)*:

a) if *d(t) *meets the two conditions related to the IMF definition (mentioned previously), an IMF is derived. Replace *x(t) *with the residual *r(t) *= *x(t) *- *d(t)*;

b) if *d(t) *is not an IMF, replace *x(t) *with *d(t)*, and

6. Repeat steps 1 to 5 until a monotonic residual, or a single maximum or minimum-residual satisfying some stopping criterion is obtained.

At the end of this process, the signal *x(t) *can be expressed as follows:(7)

where N is the number of intrinsic modes,

*r*_*N*_(*t*) denotes the final residue, which can be interpreted as the DC component of the signal,

*c*_*j*_(*t*) are he intrinsic modes, orthogonal to each other and all have zero means.

Now, Hilbert Transform can be applied to every single intrinsic function.

Freely accessible databases containing normal and abnormal EEG signals were used as resources to conduct this study. These are: Department of Epileptology at the University Hospital of Bonn, MIT and ANSI/AAMI. The sampling rate of the data is 173.61 Hz. Each signal treated having 23.6 s in duration. The time series have the spectral bandwidth of the acquisition system, which is 0.5 Hz to 85 Hz. The subsets of healthy signals have been acquired extracranially using surface EEG recordings of five healthy volunteers with eyes open and closed respectively. The other subset contains seizure activity selected from all recording sites exhibiting ictal activity. For a more detailed description of the data please refer to the manuscript [18].

Recognition of abnormal activities caused by changes in neuronal electrochemical activity was realized by EEG processing using the implemented coupling of Hilbert and EMD by MATLAB. The algorithms indicated by [19] are used to get the desired results. In the present work, EEG signals were decomposed by the EMD (2000 sifting).

Recognition in this paper was performed in three consecutive steps. First, EMD was applied to a total of 50 cases: 25 EEG signals obtained from healthy volunteers and 25 EEG signals obtained from seizure activities. The main goal of this first step was to extract the intrinsic modes in every EEG signal. Second, Hilbert Transform was applied to every intrinsic mode with the aim of tracking instantaneous frequencies and amplitudes. Finally, the weighted frequencies of counterpart intrinsic modes were statistically compared using the student's t-test [15]. The t-test assesses whether the distribution means of the two groups are statistically different from each other. The weighted frequency index of the proposed approach, when it provides a significant difference, made it possible to discriminate between healthy and seizure activities. On the other hand, using only this test for classification does not provide complete information about the type of signals being classified. Supervised Euclidean Clustering of signals has been therefore proposed, applied and evaluated in terms of sensitivity and specificity.

## Results

**Figure 2:**
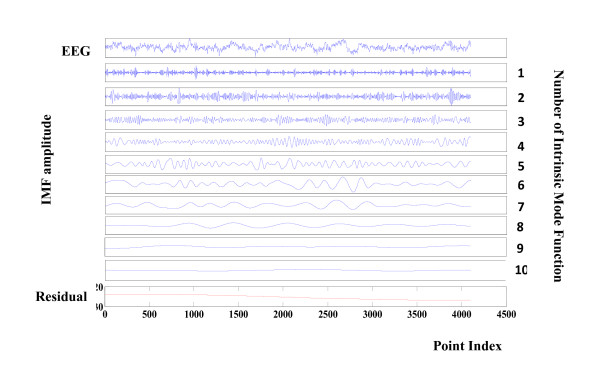
**Components of a healthy EEG signal**. Decomposition of a healthy EEG signal by EMD. The first time series is the EEG signal. The decomposition yields 10 IMF and a residual. The IMF are the time-frequency constituents or components of the EEG signal. Frequency content is ordered in a descending order (IMF1 has the highest frequency content).

Figure 2 illustrates the result of decomposition performed by EMD of a healthy EEG signal. Figure 2 shows that the first mode has a higher frequency than the second mode where modes are ordered from the highest frequency to the lowest. The main components of the EEG signal are located in the first four modes and the lower modes indicate artefact, trend and low-frequency EEG.

**Figure 3:**
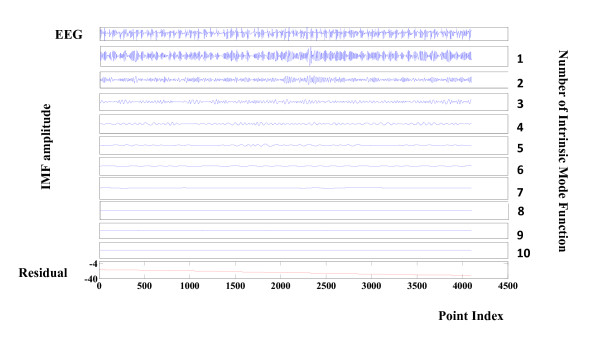
**Components of a seizure EEG signal**. Decomposition of a seizure EEG signal by EMD. The first time series is the EEG signal. The decomposition yields 10 IMF and a residual. The IMF are the time-frequency constituents or components of the EEG signal. Frequency content is ordered in a descending order. (IMF1 has the highest frequency content).

In contrast, Figure 3 shows the result of the decomposition applied to an ictal EEG signal (seizure) with the main components of the EEG signal located in the first four modes.

A visual comparison between Figure 2 and Figure 3 may lead to a qualitative discrimination. As mentioned previously, a quantitative discrimination can be applied by means of Hilbert-Huang spectrum based on local frequency/amplitude information. The following four figures indicate a comparative illustration between ictal and seizure free instantaneous frequency and amplitude.

**Figure 4:**
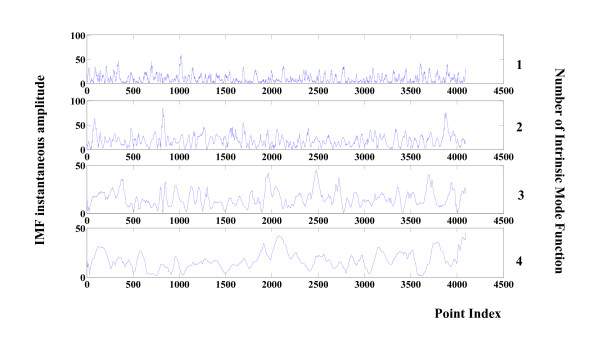
**Instantaneous amplitudes of components 1 to 4 of a healthy EEG**. Instantaneous amplitudes related to the first four components of the healthy EEG signal in shown Figure 2. Values are calculated via the Hilbert-Huang Transform.

**Figure 5:**
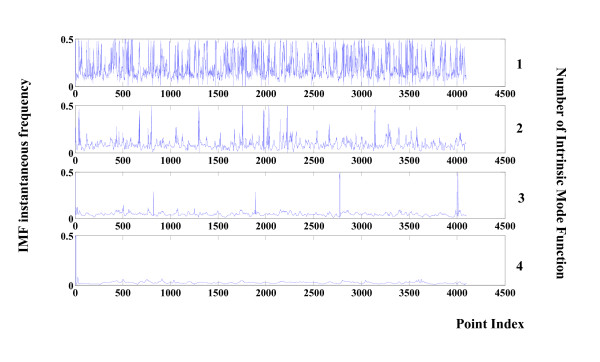
**Instantaneous frequencies of components 1 to 4 of a healthy EEG**. Instantaneous frequencies related to the first four components of the healthy EEG signal shown in Figure 2. Values are calculated via the Hilbert-Huang Transform.

The instantaneous amplitude value for the first four modes in Figure 2 (seizure free) is depicted in Figure 4. The counterpart seizure free instantaneous frequencies obtained by Hilbert Transform are presented in Figure 5.

**Figure 6:**
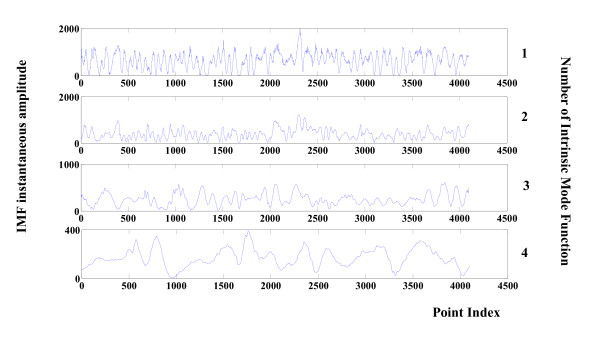
**Instantaneous amplitudes of components 1 to 4 of a seizure EEG**. Instantaneous amplitudes related to the first four components of the seizure EEG signal shown in Figure 3. Values are calculated via the Hilbert-Huang Transform.

**Figure 7:**
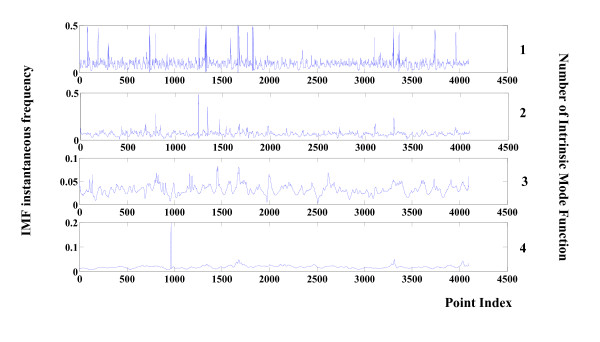
**Instantaneous frequencies of components 1 to 4 of a seizure EEG**. Instantaneous frequency related to the first four components of the seizure EEG signal shown in Figure 3. Values are calculated via Hilbert-Huang Transform.

To compare with epilepsy, the results of the frequency/amplitude analysis of an ictal signal are presented in Figures 6 and 7. Values are clearly different from those of seizure free EEG.

The criterion used for comparison in this paper is based on the previously calculated frequencies and amplitudes. It is the weighted Hilbert frequency value, which is considered in this work as the main feature upon which discrimination is based.

Table 1 contains the values of the calculated weighted frequencies for the first four modes of 10 healthy signals and 10 seizure signals (for better illustration, not all values are shown). According to the t-test, the weighted frequencies of the first four ictal and seizure free EEG intrinsic functions are significantly different (p < 0.05). A hypothesis testing with a lower p-value (p < 0.02) -high rejection region- indicates that the first three modes are highly determinant.

**Table 1:** Comparison between the weighted frequencies of the first four EMD modes

	Healthy1	Healthy2	Healthy3	Healthy4	Healthy5	Healthy6	Healthy7	Healthy8	Healthy9	Healthy10
**W.F_IMF1**	0,1676	0,1687	0,1712	0,2729	0,2475	0,1728	0,1542	0,1741	0,2453	0,1905
**W.F_IMF2**	0,076	0,0791	0,0779	0,1111	0,0912	0,0822	0,0723	0,0825	0,1082	0,0867
**W.F_IMF3**	0,0408	0,0408	0,0441	0,0592	0,0521	0,0463	0,0395	0,0456	0,0517	0,0481
**W.F_IMF4**	0,0254	0,0353	0,0265	0,0324	0,0273	0,0237	0,0184	0,0519	0,0258	0,0412

	**Seizure1**	**Seizure2**	**Seizure3**	**Seizure4**	**Seizure5**	**Seizure6**	**Seizure7**	**Seizure8**	**Seizure9**	**Seizure10**

**W.F_IMF1**	0,0875	0,1039	0,0998	0,0968	0,1145	0,1106	0,1347	0,0608	0,1284	0,0992
**W.F_IMF2**	0,0573	0,066	0,069	0,0373	0,0458	0,0447	0,0879	0,0396	0,058	0,0629
**W.F_IMF3**	0,0241	0,0333	0,0326	0,0335	0,0274	0,0389	0,0623	0,0344	0,0309	0,0326
**W.F_IMF4**	0,0319	0,0189	0,0161	0,0102	0,0194	0,0135	0,0474	0,013	0,018	0,0187

**Figure 8:**
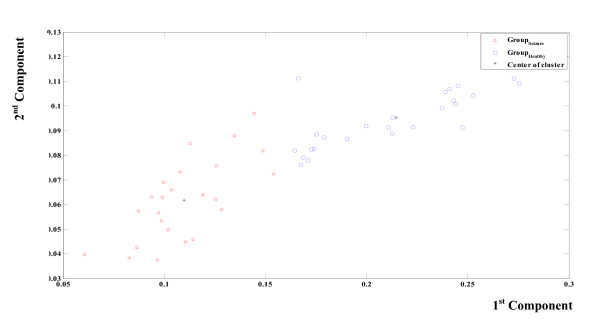
**Unsupervised Seizure Identification Using EMD**. The classification of the calculated weighted frequencies by a supervised clustering using the Euclidian distance. Every point holds the information about the weighted frequencies of the first four IMF of an EMD processed EEG signal. Two different groups of points are illustrated; the first group (circles) indicates healthy EEG. The second group (stars) indicates seizure EEG. Asterisks indicate two different centres of group. The lower one is related to the seizure group.

As shown in figure 8, the classification by a supervised clustering using the Euclidian distance has also been applied and evaluated. This figure shows two distinct groups: ictal and seizure free weighted frequencies. The groups are concentrated around two different centroids. Table 2 indicates the classification performance. The accuracy is 94%; the specificity is 96% and the sensitivity is 92%. To take the proposed method a step further, a comparison against MEMD is conducted. The same 50 signals applied to EMD have been also applied to MEMD. Four channels have been used; two identical healthy EEG channels and two identical seizure EEG channels. According to MEMD every signal is considered as a time series. The 50 time series represents two data-sets (25 healthy EEG and 25 seizure EEG). After the application of MEMD to all of the couples of the time series, the Hilbert Transform was then applied to all IMF (200) and the weighted frequency for every IMF has been calculated.

**Table 2:** Evaluation of classification performance

Parameter	Value
Accuracy	94%
Specificity	96%
Sensitivity	92%

**Figure 9:**
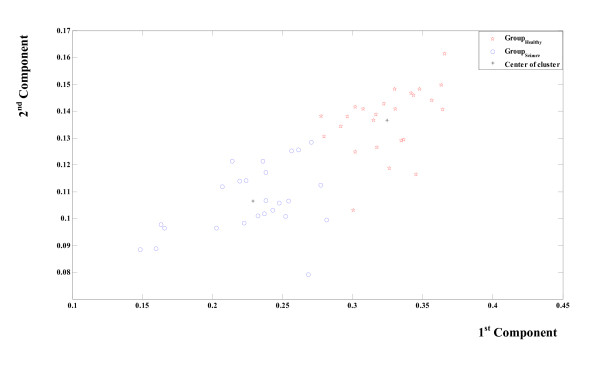
**Unsupervised Seizure Identification Using MEMD**. Figure legend text. The classification of the calculated weighted frequencies by a supervised clustering using the Euclidian distance. Every point holds the information about the weighted frequencies of the first four IMF of an MEMD processed EEG signal. Two different groups of points are illustrated; the first group (stars) indicates healthy EEG. The second group (circles) indicates seizure EEG. Asterisks indicate two different centres of group. The lower one is related to the seizure group.

Figure 9 shows the classification by a supervised clustering using the Euclidian distance applied to MEMD. The groups are once again concentrated around two different centroids (healthy and seizure). Table 3 contains the values of the calculated weighted frequencies for the first four MEMD of 10 healthy signals and 10 seizure signals (for better illustrations, not all the values are shown). Table 4 indicates the classification performance based on 50 values (25 couples of healthy and seizure signals). The obtained accuracy is 80%, the specificity is 80%, and the sensitivity is 81%.

**Table 3:** Comparison between the weighted frequencies of the first four MEMD modes

	Healthy1	Healthy2	Healthy3	Healthy4	Healthy5	Healthy6	Healthy7	Healthy8	Healthy9	Healthy10
**W.F_IMF1**	0.2195	0.3020	0.2225	0.3635	0.3568	0.2615	0.2141	0.2797	0.3302	0.2380
**W.F_IMF2**	0.1139	0.1248	0.0982	0.1498	0.1440	0.1256	0.1213	0.1304	0.1482	0.1172
**W.F_IMF3**	0.0631	0.0665	0.0659	0.0846	0.0791	0.0699	0.0625	0.0947	0.0873	0.0602
**W.F_IMF4**	0.0795	0.0603	0.0656	0.0793	0.0720	0.0682	0.0442	0.0515	0.0634	0.0567

	**Seizure1**	**Seizure2**	**Seizure3**	**Seizure4**	**Seizure5**	**Seizure6**	**Seizure7**	**Seizure8**	**Seizure9**	**Seizure10**

**W.F_IMF1**	0.1597	0.2816	0.1486	0.3175	0.3453	0.2240	0.1634	0.2542	0.2773	0.1659
**W.F_IMF2**	0.0887	0.0994	0.0883	0.1265	0.1163	0.1141	0.0977	0.1064	0.1123	0.0964
**W.F_IMF3**	0.0575	0.0603	0.0511	0.0790	0.0655	0.0617	0.0607	0.0564	0.0628	0.0554
**W.F_IMF4**	0.0329	0.0319	0.0305	0.0362	0.0340	0.0336	0.0370	0.0347	0.0341	0.0331

**Table 4:** Evaluation of MEMD classification performance

Parameter	Value
Accuracy	80%
Specificity	80%
Sensitivity	81%

## Discussion

The proposed classification approach presented in this paper needs to be further investigated as it holds a promising potential in biomedical application. It can be enhanced to be used as a computer aided diagnostic tool in which the resulting classification is used to identify seizure based on the weighted frequency to detect any abnormalities and list the diagnosis accordingly. The findings indicate also that the proposed classification was insensitive to the noise in treated signals, thus, maintaining its levels of high accuracy. Furthermore, its ability in solving the seizure classification without a model-based procedure is extremely advantageous, especially that this task is hard or even impossible. Hence, the proposed technique is a highly useful and powerful classification tool that fulfilled the independency requirement of being a self automated classification tool.

The proposed method has an advantage of instantaneous frequency/energy tracking, which is very necessary for diagnosis and outperforms techniques based on only average-values. Thus, it can also be easily used as an online technique, where classified EEG signals are decomposed and converted to the analytical form.

A hypothesis testing using the t-test with two different p-values indicates that the first three or four modes are highly determinant. Hence, the rejection region should be clearly determined before proceeding to the classification. Moreover, other statistical classification methods can complement the proposed t-test based comparison. For instance, independent component analysis and clustering techniques might give an acceptable result of seizure classification via classes' construction. In other words, if the t-test shows significant differences between the data of two subjects, one normal and the other epileptic, no one can determine which one is epileptic or normal. Furthermore, if one adds some noise to the data of one subject, and then applies the same classification method to the original data and noisy one, probably can find a significant difference between them. Hence, a different supervised classification criterion has been used.

It is worth noting that EMD has no analytical formulation; hence, our understanding of EMD comes from experimental rather than analytical results. From experimental results, it is shown that mode mixing and mode intermittency are the major obstacles to the use of EMD. Mode mixing indicates that oscillations of different time scales coexist in a given mode, or that oscillations with the same time scale have been assigned to different modes. Hence, this may lead sometimes to a misunderstanding of the real process.

The presented work reaches a higher accuracy and faster response than MEMD but a slightly lower accuracy than the work of Tzallas et al. The fact that the approach of MEMD leads to similar frequency content in the corresponding IMFs makes the classification task harder, hence the Euclidian clustering might not be sufficient. In other words, the fact that MEMD forces decomposition to have a specific pattern of IMF frequency content order leads to a complicated case of mode mixing, which is one of the disadvantages of empirical decomposition. This drawback makes Hilbert Transform to be not a very good choice to track frequency [17]. The neural networks might be in this case a good alternative.

## Conclusions

The proposed techniques in this paper are used for EEG signal processing and combined to come up with a new original tool that gives physicians the possibility to diagnose brain functionality abnormalities. The proposed system bear the potential of providing several credible benefits such as fast diagnosis, high accuracy, good sensitivity and specificity, time saving and user friendly. Furthermore, given that the main perspective of this work is to detect and solve EEG mode mixing via an elaborated signal processing solution, it may be concluded that the classification of mode mixing can be achieved using the extracted instantaneous information of every IMF, but it would be most likely a hard task if only the average value is used. Extra benefits of this proposed system include low cost, and ease of interface. All of that indicate the usefulness of the tool and its use as an efficient diagnostic tool.

## Competing interests

The authors declare that they have no competing interests.

## Authors' contributions

RO came up with the idea and proposed the design concept. EA implemented the concept and performed modifications related to the design concept. Both authors made date interpretation after analysis and both have been involved in drafting the manuscript and revising it for intellectual content. Both authors read and approved the final manuscript.
